# Development of a highly efficient protoplast regeneration and transfection protocol for enhancing CRISPR genome editing of *Brassica carinata*

**DOI:** 10.3389/fpls.2025.1604283

**Published:** 2025-09-19

**Authors:** Xueyuan Li, Misteru Tesfaye, Sjur Sandgrind, Rui Guan, Li-Hua Zhu

**Affiliations:** Department of Plant Breeding, Swedish University of Agricultural Sciences, Lomma, Sweden

**Keywords:** *Brassica carinata*, protoplast regeneration, transfection, oilseed and industrial crop, genome editing

## Abstract

*Brassica carinata* is an important oil crop with significant potential for food and industrial production. The application of the CRISPR/Cas9 genome editing tool in *B.* c*arinata* could accelerate its breeding cycle. However, no efficient DNA-free gene editing method currently exists for this species. Protoplast-based CRISPR editing presents a promising solution, though it is often challenging for many crop species. In this study, we investigated several critical factors influencing *in vitro* shoot regeneration, including genotype, sugar type, selection and combination of plant growth regulators (PGRs), and culture duration on different media throughout various stages of protoplast development. As a result, we developed a highly efficient, five-stage protoplast regeneration protocol for *B. carinata* based on specific stages of protoplast development. Key findings of this study include the requirement for high concentrations of NAA and 2,4-D in the initial medium (MI) for cell wall formation, while a lower auxin concentration relative to cytokinin was necessary for active cell division (MII). For callus growth and shoot induction, a high cytokinin-to-auxin ratio was essential (MIII), and an even higher cytokinin-to-auxin ratio was optimal for shoot regeneration (MIV). For shoot elongation, low levels of BAP and GA_3_ were sufficient (MV). Our results also demonstrated that the duration of culture on different media and maintaining appropriate osmotic pressure at the early stages were crucial for successful protoplast regeneration. With this optimized protocol, we achieved an average regeneration frequency of up to 64% and a transfection efficiency of 40% using the *GFP* marker gene. This efficient protoplast regeneration protocol is now being employed for genome editing in our lab and is expected to significantly enhance the application of the CRISPR system in both basic research and the genetic improvement of *B. carinata* over the long term.

## 1 Introduction

*Brassica carinata*, commonly known as Ethiopian mustard or locally as gomenzer, is an amphidiploid species (BBCC, 2n = 34) resulting from interspecific hybridization between *B. nigra* L. (BB, 2n = 16) and *B. oleracea* L. (CC, 2n = 18). *B. carinata* is genetically diverse and well-adapted to the highland areas of Ethiopia, where it is grown for its edible leaves and seeds, which are used for oil production. This crop is known for its exceptional heat and drought tolerance, resistance to blackleg disease, and resilience against pests like aphids and flea beetles, making it suitable for areas where other crops struggle to thrive (Taylor et al., 2010). Additionally, *B. carinata* has gained attention as a potential bioindustrial feedstock (Roslinsky et al., 2021).

Despite many *B. carinata* genotypes showing good agronomic potential, some key traits such as seed oil quality, still do not meet the requirements for commercial production. One such significant limitation is the high erucic acid content in the seed oil, which reduces its suitability for food applications. Compared to crops like rapeseed, *B. carinata* is found to be a neglected oil crop and has undergone less intensive breeding. The application of modern breeding technologies could expedite the improvement of elite cultivars or breeding lines by addressing specific limitations and making efficient use of its valuable natural genetic resources.

Genome editing using the CRISPR/Cas9 system has become increasingly popular in plant research and crop breeding (Shan et al., 2013). Most CRISPR editing in plants has been performed using tissue culture-based methods, where DNA-based or RNA-based CRISPR complexes are delivered into plant cells. DNA-based CRISPR delivery methods, such as *Agrobacterium*-mediated transformation or particle bombardment (Arora and Narula, 2017), often result in stable integration of CRISPR vectors, leading to transgenic plants. However, the integration of CRISPR complexes into the plant genome raises concerns about ongoing vector expression, increased risk of off-target mutations, and regulatory hurdles in countries with strict GMO policies (Woo et al., 2015).

PEG-mediated protoplast transient expression has emerged as a promising method for delivering CRISPR complexes into plant protoplasts. This approach allows gene editing without the integration of foreign DNA, producing desirable mutants through transient expression (Armstrong et al., 1990; Kim et al., 2017; Jiang et al., 2013; Liang et al., 2017; Woo et al., 2015). Although the PEG-protoplast method has been successful in species such as Arabidopsis (Li et al., 2013), tobacco (Gao et al., 2015), rice (Feng et al., 2013), maize (Liang et al., 2014), wheat (Zhang et al., 2016), potato (Andersson et al., 2018; Tiwari et al., 2022), rapeseed (Li et al., 2021; Moss et al., 2025), and field cress (Sandgrind et al., 2021, 2023), it remains challenging for many crops, and protoplast regeneration systems are still underdeveloped for species like *B. carinata*.

Protoplast research in *Brassica* species began in the early 1980s, but much of the initial work focused on protoplast isolation, culture, and fusion rather than regeneration. Limited research has been conducted on *B. carinata* protoplast regeneration. Early studies reported low shoot regeneration frequencies from cotyledon protoplasts (Jaiswal et al., 1990) and slightly better results from hypocotyl protoplasts (Chuong et al., 1987; Narasimhulu et al., 1992). However, the use of hypocotyls requires a large amount of plant material, making the method impractical for broader application. In contrast, leaf tissues are easier to work with and provide higher protoplast yields in species like rapeseed (Li et al., 2021; Moss et al., 2025), making them more suitable for protoplast isolation. Yet, there remains a lack of comprehensive studies on protoplast regeneration in *B. carinata*, which has been a barrier to effective CRISPR/Cas9 application for genome editing in this species.

In our study, we systematically examined the major factors influencing protoplast regeneration and transfection in *B. carinata*. We successfully developed an efficient protoplast regeneration and transfection protocol, which will significantly facilitate both basic research and crop improvement in the species and related oilseed species.

## 2 Material and method

### 2.1 Plant material

Seeds from three genotypes of *B. carinata* obtained from Ethiopia were used in this study: the advanced line S-67 x Holetta-1 (referred to as G1), cultivar Tesfa (referred to as G2), and cultivar Derash (referred to as G3). G1 and G3 were included in all experiments, while G2 was used only for genotype comparison as it showed a similar regeneration capacity as G3. The reason to choose G3 for further study was its oil content was higher than G2 according to Tesfaye et al. (2024).

### 2.2 Seed germination

Seeds were surface sterilized by soaking in 15% (v/v) calcium hypochlorite (CaCl_2_O_2_) for 20 minutes and thoroughly rinsed with sterile water. The sterilized seeds were then planted on germination medium in sterile plastic boxes. The germination medium consisted of half-strength Murashige and Skoog (MS) salts, 10 g l^-1^ sucrose, and 7 g l^-1^ Bacto agar, with a pH of 5.7. The boxes were placed in a climate-controlled chamber set to 25°C during the day and 18°C at night, with a 16-hour photoperiod and light intensity of 40 µmol m^-2^ s^-1^ provided by cool white fluorescent tubes.

### 2.3 Protoplast isolation

Protoplast isolation was performed following the protocol by Li et al. (2021). Briefly, approximately 40 fully expanded leaves were harvested from 3- to 4-week-old seedlings (3 weeks for G2 and G3, 4 weeks for G1 due to a lower growth rate. The leaves were placed on damp filter paper in a Petri dish, finely sliced using scalpels or razor blades, and incubated in plasmolysis solution (0.4 M mannitol, pH 5.7) in the dark at room temperature (RT) for 30 minutes.

After plasmolysis, the leaf pieces were incubated in 10 ml enzyme solution in the dark at RT for 14–16 hours with gentle shaking. The enzyme solution contained 1.5% (w/v) cellulase Onozuka™ R10 (Yakult Pharmaceutical Co., LTD., Tokyo, Japan), 0.6% (w/v) Macerozyme™ R10 (Yakult Pharmaceutical Co., LTD., Tokyo, Japan), 0.4 M mannitol, 10 mM MES, 0.1% (w/v) BSA, 1 mM CaCl_2_, and 1 mM β-mercaptoethanol, adjusted to pH 5.7.

Following enzymatic digestion, 10 ml of W5 solution (Menczel et al., 1981) was added to the Petri dish, which was then gently shaken in the dark at RT for 10 minutes. The protoplast solution was filtered through a 40 µm nylon mesh, and the Petri dish was washed with 20 ml of W5 solution. The filtered protoplast suspension was centrifuged at 100 g for 10 minutes. The pellet was resuspended in 10 ml of W5 solution, centrifuged again for 5 minutes at 100 g, and this process was repeated twice. The final pellet was resuspended in 5 ml of W5 solution and kept on ice in the dark for 30 minutes.

The light-green supernatant was discarded, and the protoplasts were diluted with 5–10 ml of W5 solution, depending on the size of the pellet. To count the protoplasts, 15 µl of the solution was loaded onto a hemocytometer. After centrifugation for 3 minutes at 100 g, the protoplast density was adjusted to 400 000-600–000 cells per ml using 0.5 M mannitol. An equal volume of sodium alginate solution (2.8% (w/v) sodium alginate, 0.4 M mannitol) was added and mixed gently, according to the protocol by Kiełkowska and Adamus (2012).

Approximately 600 µl of the suspension was pipetted onto calcium-agar plates (0.4 M mannitol, 2.2 g l^-1^ CaCl_2_, and 10 g l^-1^ Phyto agar) to form alginate disks. The disks were incubated at RT for 30 minutes, after which 2 ml of calcium solution (50 mM CaCl_2_, 0.4 M mannitol) was added to each disk and left to polymerize at room temperature for 1 hour. Finally, the disks were transferred to culture medium in a 6-well tissue culture plate for further development.

### 2.4 Protoplast culture and regeneration

**Figure 1:**
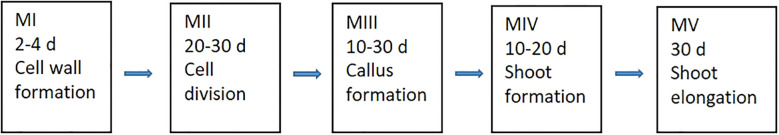
The five developmental stages of protoplasts along with their culture durations in different media (where, “d” refers to days, “M” refers to media).

Protoplasts were cultured in different media at various developmental stages, with each medium optimized as shown schematically in **Figure 1**.

At the initial stage, a liquid medium (referred to as MI) was used to promote cell wall formation in the freshly isolated protoplasts, which are highly susceptible to external damage. MI consisted of 2.18 g l^-1^ Nitsch nutrients, 10 g l^-1^ sucrose, 10 g l^-1^ glucose, 100 g l^-1^ mannitol, and 100 mg l^-1^ casein, supplemented with 0.5 mg l^-1^ 2,4-dichlorophenoxyacetic acid (2,4-D) and 0.5 mg l^-1^ α-naphthaleneacetic acid (NAA), at pH 5.7. Protoplasts were cultured in 6-well tissue culture plates, with one alginate disk per well and 2–3 ml MI medium per well. The plates were covered with aluminum foil and incubated at room temperature (RT) for 24 hours. Afterward, the aluminum foil was replaced with fiber cloth, and the plates were transferred to a climate-controlled chamber as previously described.

After 3–4 days, the MI medium was replaced with a liquid medium referred to as MII, designed to stimulate protoplast cell division and callus formation. MII was similar to MI, except the plant growth regulators (PGRs) which were replaced by 1.1 mg l^-1^ thidiazuron (TDZ) and 0.05 mg l^-1^ 2,4-D. The duration of culture in MII and its impact on protoplast regeneration were tested at this stage. During the 20–30 day culture period, the MII medium was refreshed every 5–7 days.

Once protoplast colonies formed on alginate disks, they were transferred directly to solid medium (MIII) in Petri dishes for callus formation and shoot induction. Different PGR combinations and culture durations were tested during this stage and detailed medium compositions are presented in the results section. MIII medium was renewed every 5–7 days during 10–50 days until microprotoplast calli reached 0.1-0.2 mm in diameter. All MIII media contained 0.5 mg l^-1^ AgNO_3_ and 2.5 g l^-1^ Gelrite at pH 5.7.

The microprotoplast calli were then transferred to shoot regeneration medium (MIV). Various PGR combinations were tested for shoot regeneration, with the detailed compositions provided in the results section. All MIV media contained 0.5 mg l^-1^ AgNO_3_ and 2.5 g l^-1^ Gelrite at pH 5.7.

Regenerated shoots were subsequently moved to shoot elongation medium (MV), which consisted of full-strength MS, 20 g l^-1^ sucrose, 0.05 mg l^-1^ 6-benzyladenine (BAP), 0.03 mg l^-1^ gibberellic acid (GA_3_), and 7.5 g l^-1^ agar at pH 5.7.

The elongated shoots were finally transferred to the rooting medium, containing half-strength MS, 20 g l^-1^ sucrose, 0.05 mg l^-1^ NAA, and 7.5 g l^-1^ agar at pH 5.7.

### 2.5 Protoplast transfection

Optimizing transfection efficiency is crucial for successful genome editing. Therefore, we tested different factors affecting protoplast transfection efficiency using a vector containing the green fluorescent protein (*GFP*) gene (pCW498-35S-GFiP-OcsT, 14,743 bp) (Wood et al., 2009).

Approximately 150,000 washed protoplasts of G1 were re-suspended in 200 µl of freshly prepared MMG solution (0.4 M mannitol, 15 mM MgCl_2_, 4 mM MES) in a 2 ml Eppendorf tube. The suspension was mixed with 40 µg of *GFP* vector DNA and an equal volume of freshly prepared PEG-calcium solution (25% (w/v) PEG4000, 0.4 M mannitol, 0.1 M CaCl_2_). The reaction was stopped after 15 minutes by adding 1.5 ml W5 solution and gently inverting the tube. The mixture was centrifuged at 100 g for 3 minutes, and the supernatant was immediately discarded. Transfected protoplasts were re-suspended in 1 ml MI medium, transferred to a six-well tissue culture plate, and incubated in the dark at RT. The plate was then moved to the climate-controlled chamber for further incubation.

### 2.6 Detection of GFP gene expression

To assess transfection efficiency, GFP expression in protoplasts was observed 48 hours post-transfection using a Zeiss LSM 880 Airyscan confocal laser scanning microscope.

### 2.7 Statistical analysis

Statistical analysis was conducted on the regeneration tests. Each treatment included at least three biological replicates, with each replicate consisting of 20–40 protoplast colonies. Regeneration results were recorded after 30 days of culture on MIV medium when shoots began to appear. Data were analyzed using ANOVA and Tukey’s test via Minitab (LLC) version 19.2020.1.

## 3 Results

### 3.1 Effect of genotypes on protoplast regeneration

**Figure 2:**
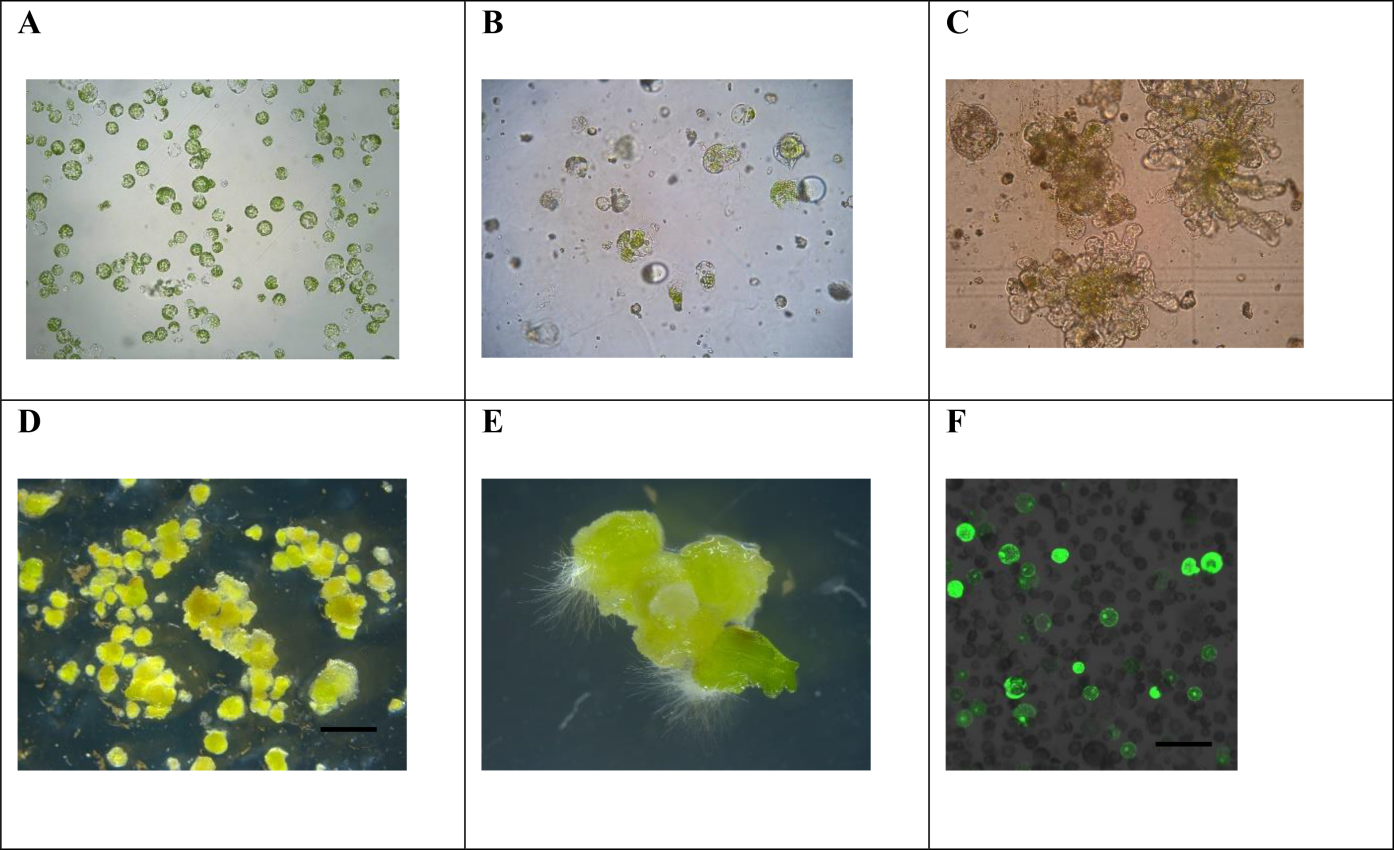
Microscopic images of different protoplast developmental stages **(A–F)** and protoplast transfection of *B. carinata*. **(A)** Freshly isolated protoplast (Day 1). **(B)** Growing protoplast cells (Day 7 or 15). **(C)** Callus growth (Day 21). **(D)** Micro protoplastcalli (on CIM medium 25d). **(E)** Shoot formed from callus (on SIM medium 10d). **(F)** GFP expression in protoplast after transfection (48h after transfection).

Three genotypes of *B. carinata* were tested in this study, with the results presented in **Table 1**. Significant differences in regeneration frequency were observed among the genotypes. Genotype 1 (G1) exhibited the highest regeneration frequency at 64%, while G2 and G3 had lower regeneration frequencies of 31% and 26%, respectively. A figure panel is presented in **Figure 2**, representing different development stages of protoplast culture of *B. carinata*.

**Table 1:** Effect of genotype on protoplast regeneration efficiency.

Genotype	Regeneration efficiency (%)
G1	0.64 a
G2	0.31 b
G3	0.26 b

MI composition: Full Nitsch, 10 g l^-1^ sucrose, 10 g l^-1^ glucose, 100 g l^-1^ mannitol, 100 mg l^-1^ casein, 0.5 mg l^-1^ NAA, 0.5 mg l^-1^ 2,4-D. MII: Full Nitsch, 10 g l^-1^ sucrose, 10 g l^-1^ glucose, 100 g l^-1^ mannitol, 100 mg l^-1^ casein, 1.1 mg l^-1^ TDZ, 0.05 mg l^-1^ 2,4-D. MIII: Full MS, 30 g l^-1^ sucrose, 50 g l^-1^ mannitol, 2.2 mg l^-1^ TDZ, 0.05 mg l^-1^ NAA, 0.5 mg l^-1^. MIV: Full MS, 30 g l^-1^ sucrose, 2.2 mg l^-1^ TDZ, 0.05 mg l^-1^ NAA, 0.5 mg l^-1^. Values followed by different letters were statistically different at *p*=0.05.

### 3.2 Effect of MII medium composition and culture duration on protoplast regeneration

#### 3.2.1 Effect of mannitol concentration in MII on protoplast regeneration

The initial phase of protoplast (**Figure 2A**) development involves cell wall formation, which is crucial for rapid cell division (**Figure 2B**). The composition of MI and MII media, particularly nutrients, plant growth regulators (PGRs), and mannitol concentrations, plays a critical role in promoting cell wall and callus formation (**Figures 2C, D**). In this study, we used the same MI and MII media as reported for other oil crops (Li et al., 2021; Sandgrind et al., 2021), and they proved to work well for *B. carinata*.

Mannitol is essential for maintaining osmotic pressure in protoplasts during early development when the cell wall is not fully formed. However, excessive mannitol concentrations can inhibit cell division. We tested the effect of mannitol in MII medium by including three concentrations of mannitol (0, 50, 100 g l^-1^). The results showed that only a concentration of 100 g l^-1^ mannitol supported shoot regeneration (**Figure 2E**) in G1 and G3 (**Table 2**). This indicates that high mannitol concentrations are crucial for shoot regeneration during this stage, as the cell wall remains underdeveloped.

**Table 2:** Effect of Mannitol in MII medium on protoplast regeneration efficiency (%).

Genotype	G1	G3
Mannitol 100 g l^-1^	0.64 a	0.24 a
Mannitol 50 g l^-1^	0.0 b	0.0 b
Mannitol 0 g l^-1^	0.0 b	0.0 b

MII composition: 2.18 g l^-1^ Nitsch medium, 10 g l^-1^ sucrose, 10 g l^-1^ glucose, 100 mg l^-1^ casein, 1.1 mg l^-1^ TDZ, 0.05 mg l^-1^ 2,4-D. Other media as follows: MI: 2.18 g l^-1^ Nitsch medium, 10 g l^-1^ sucrose, 10 g l^-1^ glucose, 100 g l^-1^ mannitol, 100 mg l^-1^ casein, 0.5 mg l^-1^ NAA, 0.5 mg l^-1^ 2,4-D. MIII: Full MS, 30 g l^-1^ sucrose, 50 g l^-1^ mannitol, 2.2 mg l^-1^ TDZ, 0.05 mg l^-1^ NAA, 0.5 mg l^-1^ AgNO_3_. MIV: Full MS, 30 g l^-1^ sucrose, 2.2 mg l^-1^ TDZ, 0.05 mg l^-1^ NAA. Values followed by the same letter were not statistically different at *p*=0.05.

#### 3.2.2 Effect of culture duration in MII on protoplast regeneration

The effect of culture duration in MII medium on protoplast regeneration was assessed (**Table 3**). The data indicated that culture duration had a significant impact on regeneration frequency in *B. carinata*. For G1, the optimal culture duration in MII for protoplast regeneration was 25 to 30 days, resulting in the highest regeneration frequency, whereas for G3, 20 to 30 days was optimal. Longer culture durations (over 40 days) led to a sharp decline in regeneration frequency for both genotypes.

**Table 3:** Effect of culture duration in MII medium on protoplast regeneration efficiency (%).

Day	20	25	30	40	50
G1	0.10 c	0.57 a	0.62 a	0.46 b	0.16 c
G3	0.25 a	0.20 a	0.30 a	0.14 b	0.02 c

MII composition: 2.18 g l^-1^ Nitsch medium, 10 g l^-1^ sucrose, 10 g l^-1^ glucose, 100 g l^-1^ mannitol, 100 mg l^-1^ casein, 1.1 mg l^-1^ TDZ, 0.05 mg l^-1^ 2,4-D. MI: 2.18 g l^-1^ Nitsch medium, 10 g l^-1^ sucrose, 10 g l^-1^ glucose, 100 g l^-1^ mannitol, 100 mg l^-1^ casein, 0.5 mg l^-1^ NAA, 0.5 mg l^-1^ 2,4-D. MIII: Full MS, 30 g l^-1^ sucrose, 50 g l^-1^ mannitol, 2.2 mg l^-1^ TDZ, 0.05 mg l^-1^ NAA. MIV: Full MS, 30 g l^-1^ sucrose, 2.2 mg l^-1^ TDZ, 0.05 mg l^-1^ NAA, 0.5 mg l^-1^. Values followed by different letters were statistically different at *p*=0.05.

There were clear differences in regeneration frequency between G1 and G3 under different culture durations. Although both genotypes followed a similar trend, G1 exhibited slower growth, requiring a longer culture duration compared to G3. This slower growth in G1 suggests that if protoplasts are transferred to MIII too early, the callus may be too small to continue growing.

### 3.3 Impact of MIII medium on protoplast regeneration

Low regeneration frequency in protoplast culture is a common challenge for many crops. To enhance regeneration frequency in *B. carinata*, we investigated several critical factors, including the presence of mannitol, PGR combinations and the effect of culture duration in MIII medium.

#### 3.3.1 Effect of mannitol concentration in MIII medium on protoplast regeneration

Our results demonstrate that the presence of mannitol in the MIII medium remains crucial for protoplast regeneration (**Table 4**). Compared to the treatment with 50 g l^-1^ mannitol in the MIII medium, the absence of mannitol resulted in a significant decrease in protoplast regeneration frequency.

**Table 4:** Effect of Mannitol in MIII medium on protoplast regeneration efficiency (%).

Genotype	G1	G3
Mannitol 50 g l^-1^	0.64 a	0.24 a
Mannitol 0 g l^-1^	0.11 b	0.05 b

MIII composition: Full MS, 30 g l^-1^ Sucrose, 2.2 mg l^-1^ TDZ, 0.05 mg l^-1^. Other media as follows: MI: 2.18 g l^-1^ Nitsch medium, 10 g l^-1^ sucrose, 10 g l^-1^ glucose, 100 g l^-1^ mannitol, 100 mg l^-1^ casein, NAA 0.5 mg l^-1^, 2,4-D 0.5 mg l^-1^. MII: 2.18 g l^-1^ Nitsch medium, 10 g l^-1^ sucrose, 10 g l^-1^ glucose, 100 g l^-1^ mannitol, 100 mg l^-1^ casein, TDZ 1.1 mg l^-1^, 2,4-D 0.05 mg l^-1^. MIV: Full MS, sucrose 30 g l^-1^, 2.2 mg l^-1^ TDZ, NAA 0.05 mg l^-1^. Values followed by different letters were statistically different at *p*=0.05.

#### 3.3.2 Effect of PGR combinations in MIII on protoplast regeneration

After development of cell walls the protoplasts would undergo rapid cell division and form callus. The composition of MIII, especially PGR combinations, was crucial for promoting callus formation. We tested two PGR combinations in two genotypes and the results showed while callus cultured with 2.2 mg l^-1^ TDZ and 0.05 mg l^-1^ NAA grew faster than with 1.1 mg l^-1^ TDZ and 0.05 mg l^-1^ NAA, no significant differences were observed in regeneration frequency between these two media for both genotypes (**Table 5**).

**Table 5:** Effect of PGR combination in MIII medium on protoplast regeneration efficiency (%).

Genotype	G1	G3
TDZ 1.1 mg l^-1^ NAA 0.05 mg l^-1^	0.64 a	0.24 a
TDZ 2.2 mg l^-1^ NAA 0.05 mg l^-1^	0.62 a	0.27 a

MIII composition: Full MS. Other media as follows: MI composition: 2.18 g l^-1^ Nitsch medium, 10 g l^-1^ sucrose, 10 g l^-1^ glucose, 100 g l^-1^ mannitol, 100 mg l^-1^ casein, 0.5 mg l^-1^ NAA, 0.5 mg l^-1^ 2,4-D. MII: 2.18 g l^-1^ Nitsch medium, 10 g l^-1^ sucrose, 10 g l^-1^ glucose, 100 g l^-1^ mannitol, 100 mg l^-1^ casein, 1.1 mg l^-1^ TDZ, 0.05 mg l^-1^ 2,4-D. MIV: Full MS, 30 g l^-1^ sucrose, 2.2 mg l^-1^ TDZ, 0.05 mg l^-1^ NAA. Values followed by different letters were statistically different at *p*=0.05.

#### 3.3.3 Effect of culture duration in MIII medium on protoplast regeneration

In the MIII medium, protoplasts undergo regeneration induction. We investigated the effect of culture durations in MIII to improve regeneration frequency. Data in **Table 6** indicate that the duration can range from 10 to 30 days, with the optimal time being around 20 days. However, when the culture time exceeds 40 days, the regeneration frequency significantly decreases (**Table 6**).

**Table 6:** Impact of culture duration in MIII on protoplast regeneration efficiency (%).

Day	10	15	20	25	30	40
G1	0.40 c	0.53 a	0.61 b	0.59 b	0.50 a	0.16 d
G3	0.15 b	0.28 a	0.23 a	0.18 b	0.19 b	0.06 c

MI composition: 2.18 g l^-1^ Nitsch medium, 10 g l^-1^ sucrose, 10 g l^-1^ glucose, 100 g l^-1^ mannitol, 100 mg l^-1^ casein, 0.5 mg l^-1^ NAA, 0.5 mg l^-1^ 2,4-D. MII: 2.18 g l^-1^ Nitsch medium, 10 g l^-1^ sucrose, 10 g l^-1^ glucose, 100 g l^-1^ mannitol, 100 mg l^-1^ casein, 1.1 mg l^-1^ TDZ, 0.05 mg l^-1^ 2,4-D. MIII: Full MS, 30 g l^-1^ sucrose, 50 g l^-1^ mannitol, 2.2 mg l^-1^ TDZ, 0.05 mg l^-1^ NAA. MIV: Full MS, 30 g l^-1^ sucrose, 2.2 mg l^-1^ TDZ, 0.05 mg l^-1^ NAA, 0.5 mg l^-1^ AgNO_3_, 2.5g l^-1^ Gelrite. Values followed by different letters were statistically different at *p*=0.05.

### 3.4 Effect of MIV medium on protoplast regeneration

Shoots start to regenerate from protoplasts cultured in MIV. To achieve higher regeneration frequency, we investigated key factors such as sugar type and PGR combination in MIV medium for their impact on shoot regeneration.

#### 3.4.1 Effect of sugar type in MIV medium on protoplast regeneration

The impact of different sugar types on protoplast regeneration was evaluated, and the results are shown in **Table 7**. Sucrose produced a significantly higher regeneration frequency compared to glucose, indicating that sucrose is a more suitable carbon source for *B. carinata* protoplast regeneration. Furthermore, 30 g l^-1^ sucrose resulted in a higher regeneration frequency than 20 g l^-1^ sucrose when using the same PGR combination.

**Table 7:** Effect of sugar in MIV medium on protoplast regeneration efficiency (%).

Sugar and PGR combination	Regeneration efficiency		Sugar and PGR combination	Regeneration efficiency	
	G1	G3		G1	G3
Sucrose 20 g l^-1^ TDZ 2.2 mg l^-1^ NAA 0.1 mg l^-1^	0.50 a	0.12 b	Glucose 10 g l^-1^ TDZ 1.1 mg l^-1^	0.01 d	0.00 d
Sucrose 20 g l^-1^ TDZ 2.2 mg l^-1^ NAA 0.5 mg l^-1^	0.55 a	0.26 a	Glucose 20 g l^-1^ TDZ 2.2 mg l^-1^	0.06 d	0.01 d
Sucrose 30 g l^-1^ TDZ 2.2 mg l^-1^ NAA 0.1 mg l^-1^	0.62 b	0.22 a	Glucose 10 g l^-1^ TDZ 2.2 mg l^-1^ NAA 0.05 mg l^-1^	0.26 c	0.04 d
Sucrose 30 g l^-1^ TDZ 2.2 mg l^-1^ NAA 0.5 mg l^-1^	0.56 a	0.26 a	Glucose 20 g l^-1^ TDZ 2.2 mg l^-1^ NAA 0.05 mg l^-1^	0.23 c	0.10 c

MIV composition: Full MS, 0.5 mg l^-1^. Other media as follows: MI: 2.18 g l^-1^ Nitsch medium, 10 g l^-1^ sucrose, 10 g l^-1^ glucose, 100 g l^-1^ mannitol, 100 mg l^-1^ casein, 0.5 mg l^-1^ NAA, 0.5 mg l^-1^ 2,4-D. MII: 2.18 g l^-1^ Nitsch medium, 10 g l^-1^ sucrose, 10 g l^-1^ glucose, 100 g l^-1^ mannitol, 100 mg l^-1^ casein, 1.1 mg l^-1^ TDZ, 0.05 mg l^-1^ 2,4-D. MIII: Full MS, 30 g l^-1^ sucrose, 50 g l^-1^ mannitol, 2.2 mg l^-1^ TDZ, 0.05 mg l^-1^ NAA, 0.5 mg l^-1^. Values followed by different letters were statistically different at *p*=0.05.

This trend was observed in both genotypes, G1 and G3, though G3 also showed high regeneration frequency with 20 g l^-1^ sucrose. These findings align with our previous research, which demonstrated that sucrose enhances protoplast cell division (Li et al., 2021).

#### 3.4.2 Effect of PGR combinations in MIV medium on protoplast regeneration

The effect of different PGR combinations on protoplast regeneration in *B. carinata* was also investigated. Results showed that both G1 and G3 followed a similar trend, though some variation was observed among treatments. The regeneration efficiency was much higher in all media having TDZ in combination with NAA rather than TDZ alone in both G1 and G3 (**Table 8**). Notably, BAP was not effective for *B. carinata* protoplast regeneration, contrary to findings in other crops where BAP is commonly used for shoot regeneration. This highlights the unique response of *B. carinata* to PGRs compared to other species (**Table 8**).

**Table 8:** Impact of PGR Combinations in MIV Medium on Protoplast Regeneration Efficiency (%) in G1 and G3.

PGR combination	Regeneration efficiency		PGR combination	Regeneration efficiency G1 G3	
	G1	G3		G1	G3
TDZ 1.1 mg l^-1^ NAA 0.05 mg l^-1^	0.59 a	0.13 a	TDZ 0.5 mg l^-1^	0.12 c	0.12 c
TDZ 2.2 mg l^-1^ NAA 0.05 mg l^-1^	064 a	0.23 a	TDZ 1.1 mg l^-1^	0.12 c	0.06 c
TDZ 2.2 mg l^-1^ NAA 0.1 mg l^-1^	0.61 a	0.22 a	TDZ 2.2 mg l^-1^	0.15 c	0.06 c
TDZ 2.2 mg l^-1^ NAA 0.5 mg l^-1^	0.55 b	0.26 b	BAP 1.0 mg l^-1^ IBA 0.1 mg l^-1^	0.06 d	0.04 d

MIV composition: Full MS, 30 g l^-1^ Sucrose, 0.5 mg l^-1^. Other media as follows: MI: 2.18 g l^-1^ Nitsch medium, 10 g l^-1^ sucrose, 10 g l^-1^ glucose, 100 g l^-1^ mannitol, 100 mg l^-1^ casein, 0.5 mg l^-1^ NAA, 0.5 mg l^-1^ 2,4-D. MII: 2.18 g l^-1^ Nitsch medium, 10 g l^-1^ sucrose, 10 g l^-1^ glucose, 100 g l^-1^ mannitol, 100 mg l^-1^ casein, 1.1 mg l^-1^ TDZ, 0.05 mg l^-1^ 2,4-D. MIII: Full MS, sucrose 30 g l^-1^, 50 g l^-1^ mannitol, 2.2 mg l^-1^ TDZ, 0.05 mg l^-1^ NAA. Values followed by different letters were statistically different at *p*=0.05.

### 3.5 Shoot elongation and rooting

Shoots regenerated from protoplast calli could grow well and elongate quickly on the solid medium V (MV). The elongated shoots could be rooted on the rooting medium and roots appeared in about 10 days. Sometimes, elongated shoots could form roots directly on the MV medium after 3 weeks. The rooted plantlets are ready for planting in soil for further evaluation, if needed.

### 3.6 Protoplast Transfection Efficiency

To assess the efficiency of protoplast transfection in *B. carinata* using the optimized protoplast regeneration protocol, we transfected protoplasts with a transformation vector containing the *GFP* gene. Transfection efficiencies was about 40%, as determined by the presence of intact protoplasts exhibiting GFP fluorescence (**Figure 2F**). This was achieved using 25% (w/v) PEG4000 and 40 µg of vector DNA. These results indicate that a substantial proportion of the protoplasts can express the transgene for an extended period, suggesting that the transfection protocol is effective for *B. carinata* under the current culture conditions.

## 4 Discussion

Protoplasts are plant cells lacking a cell wall but containing all other cellular components. With the right conditions, these cells can differentiate and eventually form callus and shoots. This process is complex, requiring specific culture conditions at each stage of development. To achieve high regeneration frequency, it is crucial to optimize all physical, chemical, and biological factors. In this study, we employed a 5-step strategy for protoplast culture in *B. carinata*, which resulted in a high regeneration frequency.

The first step in protoplast culture is the formation of a cell wall around the protoplast membrane. This process begins within a few hours after protoplast isolation and can take several days to complete (Kartha et al., 1974). Protoplast necrosis often occurs during this period if culture conditions are suboptimal, making the composition of the MI medium critical for protoplast survival. Previous research has shown that 2,4-D is essential for maintaining protoplast viability and inducing cell division (Glimelius, 1984). Our earlier studies confirmed that a combination of 0.5 mg/L 2,4-D and 0.5 mg/L NAA was effective for protoplast regeneration in rapeseed and *Lepidium campestre* (Li et al., 2021; Sandgrind et al., 2021). This same PGR combination yielded excellent results in *B. carinata*. However, it is important to note that prolonged culture in MI (over 7 days) negatively impacted cell division, as seen in our previous studies (Li et al., 2021).

Once the cell wall is reformed, protoplasts undergo rapid mitotic division, during which cytokinin is essential as it promotes cell division. Since MI contains a high auxin concentration (0.5 mg/L 2,4-D and 0.5 mg/L NAA), it must be replaced with a medium containing a higher cytokinin-to-auxin ratio to stimulate cell division at this stage and later regeneration. In this study, we used MII medium, which had 1.1 mg/L TDZ to 0.05 mg/L 2,4-D, and achieved satisfactory regeneration results.

The third step involved transferring protoplasts from liquid medium (MII) to solid medium (MIII) to promote callus growth. A certain callus size is required before they were separated for shoot regeneration to occur. Two treatments were important in this step: (1) releasing protoplasts from the alginate gel, which otherwise would restrict callus growth, and (2) reducing the mannitol concentration in the medium from 100 g l^-1^ to 50 g l^-1^. Mannitol acts as an osmotic agent, which is critical at the early stage as freshly isolated protoplasts need osmotic protection until cell walls develop (Kao and Seguin-Swartz, 1987). The appropriate osmotic pressure during protoplast isolation and culture is required to replace the cell wall’s role as protoplast protector. A suitable osmotic pressure will ensure the protoplasts in viable form and status (Mastuti and Rosyidah, 2019). Once the cell wall is formed, osmolarity must be gradually reduced to support normal growth. However, removing mannitol too early would lead to cell death, while removing it too late would negatively impact regeneration by creating an unsuitable environment that hinders nutrient and water uptake. Our approach of starting with 100 g l^-1^ mannitol in MI and MII, then reducing it to 50 g l^-1^ in MIII before removing it completely in subsequent media, proved to enhance protoplast regeneration.

After 20–30 days on the MIII medium, the callus should be transferred to MIV medium, which has a higher cytokinin-to-auxin ratio to promote shoot regeneration. We tested several PGR combinations, all of which resulted in high regeneration frequencies. Notably, auxin was essential in the medium, as treatments lacking NAA significantly hindered callus regeneration. G1 showed significantly higher regeneration efficiency than G3 in most of the PGR combinations, supporting that plant regeneration is genotype-dependent even within the same species.

Shoots typically emerged from callus within 10 days on MIV medium. Once shoots appeared, they needed to be transferred to MV medium immediately, as the high PGR concentrations in MIV would inhibit further shoot development. Delayed transfer resulted in hyperhydricity and formation of smaller and weak buds, while vigorous shoots developed within 2 to 3 weeks on the MV medium.

We also investigated the effect of culture duration in MII and MIII media on protoplast regeneration. The rationale for these tests was based on the understanding that regeneration induction should occur at the appropriate developmental stage. Prolonged culture in suboptimal media can lead to callus aging, reducing its ability to regenerate. Our results indicated that culture duration in MII and MIII was crucial and should be adjusted according to the growth characteristics of different genotypes.

In conclusion, we have developed an efficient protoplast regeneration and transfection protocol through optimizing crucial factors such as PGR, sugar and culture duration. For initial protoplast cultures, NAA and 2,4-D are crucial, while the optimal concentration combinations of NAA and TDZ are decisive for callus formation and shoot regeneration. It should be borne in mind that TDZ at 1.1 or 2.2. resulted in similar results in shoot regeneration in *B. carinata* shown in this study, which is different from the results obtained in other plant species including rapeseed and field cress where the concentration 2.2 mg/l or above worked better for shoot regeneration (Zhu et al., 2001; Li et al., 2010, 2021; Sandgrind et al., 2021). As a cytokinin-like compound, TDZ has been shown to be highly efficient in shoot induction during genetic transformation in many plant species, while the species response to this compound may differ (Cappelletti et al., 2016; Li et al., 2010; Zhu et al., 2001). Culture duration at different developmental stages are also important for protoplast regeneration. Moreover, we showed also a clear difference in regeneration capacity among different genotypes. This optimized protocol is now being applied for genome editing in *B. carinata* in our lab. We believe that the availability of this protocol will facilitate the generation of transgene-free mutants through CRISPR RNP genome editing for crop improvement. In some regions, these transgene-free mutants are not considered genetically modified (GM), and we anticipate that they will increasingly be accepted as non-GM worldwide.

## Data Availability

The raw data supporting the conclusions of this article will be made available by the authors, without undue reservation.

## References

[B1] Andersson M. Turesson T. Olsson N. Fält A.-S. Ohlsson P. Gonzalez M. N. . (2018). Genome editing in potato via CRISPR-Cas9 ribonucleoprotein delivery. Physiol. Plant 164, 378–384. doi: 10.1111/ppl.12731, PMID: 29572864

[B2] Armstrong C. L. Petersen W. L. Buchholz W. G. Bowen B. A. Sulc S. L. (1990). Factors affecting PEG-mediated stable transformation of maize protoplasts. Plant Cell Rep. 9, 335–339. doi: 10.1007/BF00232864, PMID: 24226946

[B3] Arora L. Narula A. (2017). Gene editing and crop improvement using CRISPR-Cas9 system. Front. Plant Sci. 8, 1932. doi: 10.3389/fpls.2017.01932, PMID: 29167680 PMC5682324

[B4] Cappelletti R. Sabbadini S. Mezzetti B. (2016). The use of TDZ for the efficient *in vitro* regeneration and organogenesis of strawberry and blueberry cultivars. Sci. Hortic. 207, 117–124. doi: 10.1016/j.scienta.2016.05.016

[B5] Chuong P. V. Pauls K. P. Beversdorf W. D. (1987). Protoplast culture and plant regeneration from Brassica carinata. Braun. Plant Cell Rep. 6, 67–69. doi: 10.1007/BF00269742, PMID: 24248453

[B6] Feng Z. Zhang B. Ding W. Liu X. Yang D.-L. Wei P. . (2013). Efcient genome editing in plants using a CRISPR/Cas system. Cell Res. 23, 1229–1232. doi: 10.1038/cr.2013.114, PMID: 23958582 PMC3790235

[B7] Gao J. Wang G. Ma S. Xie X. Wu X. Zhang X. . (2015). CRISPR/Cas9-mediated targeted mutagenesis in *Nicotiana tabacum*. Plant Mol. Biol. 87, 99–110. doi: 10.1007/s11103-014-0263-0, PMID: 25344637

[B8] Glimelius K. (1984). High growth rate and regeneration capacity of hypocotyl protoplasts in some Brassicaceae. Physiol. Plant 61, 38–44. doi: 10.1111/j.1399-3054.1984.tb06097.x

[B9] Jaiswal S. K. Hammatt N. Bhojwani S. S. Cocking E. C. Davey M. R. (1990). Plant regeneration irom cotymaon protoplasts of *Brassica carinata*. Plant Cell Tissue Organ Cult. 22, 159–165. doi: 10.1007/BF00033630

[B10] Jiang W. Zhou H. Bi H. Fromm M. Yang B. Weeks D. P. (2013). Demonstration of CRISPR/Cas9/sgRNA-mediated targeted gene modification in Arabidopsis, tobacco, sorghum and rice. Nucleic Acids Res. 41, 1–12. doi: 10.1093/nar/gkt780, PMID: 23999092 PMC3814374

[B11] Kao H. M. Seguin-Swartz G. (1987). Study of factors affecting the culture of *Brassica napus* L. and *B. juncea* Coss. mesophyll protoplasts. Plant cell Tissue Organ Cult. 10, 79–90. doi: 10.1007/BF00035906

[B12] Kartha K. K. Michayluk M. R. Kao K. N. Gamborg O. L. Constabel F. (1974). Callus formation and plant regeneration from mesophyll protoplasts of rape plants (*Brassica napus* L. cv. Zephyr). Plant Sci. Lett. 3, 265–271. doi: 10.1016/0304-4211(74)90097-2

[B13] Kiełkowska A. Adamus A. (2012). An alginate-layer technique for culture of *Brassica oleracea* L. protoplasts. In Vitro Cell. Dev. Biol. Plant 48, 265–273. doi: 10.1007/s11627-012-9431-6, PMID: 22593638 PMC3337407

[B14] Kim H. Kim S. T. Ryu J. Kang B. C. Kim J. S. Kim S. G. (2017). CRISPR/Cpf1-mediated DNA-free plant genome editing. Nat. Commun. 8, 14406. doi: 10.1038/ncomms14406, PMID: 28205546 PMC5316869

[B15] Li J. Norville J. E. Aach J. McCormack M. Zhang D. Bush J. . (2013). Multiplex and homologousrecombination–mediated genome editing in Arabidopsis and *Nicotiana benthamiana* using guide RNA and Cas9. Nat. Biotechnol. 31, 688–691. doi: 10.1038/nbt.2654, PMID: 23929339 PMC4078740

[B16] Li X. Ahlman A. Yan X. Lindgren H. Zhu L.-H. (2010). Genetic transformation of the oilseed crop *Crambe abyssinica*. Plant Cell Tiss Organ Cult. 100, 149–156. doi: 10.1007/s11240-009-9630-y

[B17] Li X. Sandgrind S. Moss O. Guan R. Ivarson E. Wang E. S. . (2021). Efficient protoplast regeneration protocol and CRISPR/Cas9-mediated editing of glucosinolate transporter (*GTR*) genes in rapeseed (*Brassica napus* L.). Front. Plant Sci. 12. doi: 10.3389/fpls.2021.680859, PMID: 34305978 PMC8294089

[B18] Liang Z. Chen K. Li T. Zhang Y. Wang Y. Zhao Q. . (2017). Efficient DNA-free genome editing of bread wheat using CRISPR/Cas9 ribonucleoprotein complexes. Nat. Commun. 8, 14261. doi: 10.1038/ncomms14261, PMID: 28098143 PMC5253684

[B19] Liang Z. Zhang K. Chen K. Gao C. (2014). Targeted mutagenesis in Zea mays using TALENs and the CRISPR/Cas system. J. Genet. Genomics 41, 63–68. doi: 10.1016/j.jgg.2013.12.001, PMID: 24576457

[B20] Mastuti R. Rosyidah M. (2019). *In Vitro* Enzymatic Isolation of Protoplasts fromTissuesofthe Medicinal Plant *Physalis angulata* L. AIP Conf. Proc. 020002:1-5. doi: 10.1063/1.5061838

[B21] Menczel L. Nagy F. Kiss Z. Maliga P. (1981). Streptomycin resistant and sensitive hybrids of Nicotiana tabacum + Nicotiana knightiana: correlation of resistance to *N. tabacum* plastids. Theor. Appl. Genet. 59, 191–195. doi: 10.1007/BF00264975, PMID: 24276446

[B22] Moss O. Li X. Wang E. S. Kanagarajan S. Guan R. Ivarson E. . (2025). Knockout of *BnaX.SGT.a* caused significant sinapine reduction in transgene-free rapeseed mutants generated by protoplastbased CRISPR RNP editing. Front. Plant Sci. 15. doi: 10.3389/fpls.2024.1526941, PMID: 39840369 PMC11746026

[B23] Narasimhulu S. B. Kirti P. B. Prakash S. Chopra V. L. (1992). Rapid and efficient plant regeneration from hypocotyl protoplasts of *Brassica carinata*. Plant Ceil Rep. 11, 159–162. doi: 10.1007/BF00232171, PMID: 24213551

[B24] Roslinsky V. Falk K. C. Gaebelein R. Mason A. S. Eynck C. (2021). Development of B. carinata with super-high erucic acid content through interspecific hybridization. Theoretical Applied Genetics 134, 3167–3181., PMID: 34269830 10.1007/s00122-021-03883-2 PMC8440251

[B25] Sandgrind S. Li X. Ivarson E. Ahlman A. Zhu L.-H. (2021). Establishment of an efficient protoplast regeneration and transfection protocol for field cress (*Lepidium campestre*). Front. Genome Ed. 3. doi: 10.3389/fgeed.2021.757540, PMID: 34870274 PMC8635052

[B26] Sandgrind S. Li X. Ivarson E. Wang E. S. Guan R. Kanagarajan S. . (2023). Improved fatty acid composition of field cress (*Lepidium campestre*) by CRISPR/Cas9-mediated genome editing. Front. Plant Sci. 14. doi: 10.3389/fpls.2023.1076704, PMID: 36755695 PMC9901296

[B27] Shan Q. Wang Y. Li J. Zhang Y. Chen K. Liang Z. . (2013). Targeted genome modification of crop plants using a CRISPR-Cas. system. Nat. Biotechnol. 31, 686–688. doi: 10.1038/nbt.2650, PMID: 23929338

[B28] Taylor D. C. Falk K. C. Palmer C. D. Hammerlindl J. Babic V. Mietkiewska E. . (2010). Brassica carinata–a new molecular farming platform for delivering bio-industrial oil feedstocks: case studies of genetic modifications to improve very long-chain fatty acid and oil content in seeds. Biofuels, Bioproducts Biorefining 4 (5), 538–561.

[B29] Tesfaye M. Feyissa T. Hailesilassie T. Wang E. S. Kanagarajan S. Zhu L.-H. (2024). Rapid and non-destructive determination of fatty acid profile and oil content in diverse Brassica carinata germplasm using fourier-transform near-infrared spectroscopy. Processes 12, 244. doi: 10.3390/pr12020244

[B30] Tiwari J. K. Buckseth T. Challam C. Zinta R. Bhatia N. Dalamu D. . (2022). CRISPR/Cas genome editing in potato: current status and future perspectives. Front. Genet. 13. doi: 10.3389/fgene.2022.827808, PMID: 35186041 PMC8849127

[B31] Woo J. W. Kim J. Kwon S. I. Corvalan C. Cho S. W. Kim H. . (2015). DNA-free genome editing in plants with preassembled CRISPRCas9 ribonucleoproteins. Nat. Biotechnol. 33, 1162–1164. doi: 10.1038/nbt.3389, PMID: 26479191

[B32] Wood C. C. Petrie J. R. Shrestha P. Mansour M. P. Nichols P. D. Green A. G. . (2009). A leaf-based assay using interchangeable design principles to rapidly assemble multistep recombinant pathways. Plant Biotechnol. J. 7, 914–924. doi: 10.1111/j.1467-7652.2009.00453.x, PMID: 19843252

[B33] Zhang Y. Liang Z. Zong Y. Wang Y. Liu J. Chen K. . (2016). Efficient and transgene-free genome editing in wheat through transient expression of CRISPR/Cas9 DNA or RNA. Nat. Commun. 7, 12617. doi: 10.1038/ncomms12617, PMID: 27558837 PMC5007326

[B34] Zhu L.-H. Holefors A. Ahlman A. Xue Z. Welander M. (2001). Transformation of the apple rootstock M.9:29 with the *rolB* gene and its influence on rooting and growth. Plant Sci. 160, 433–439. doi: 10.1016/S0168-9452(00)00401-5, PMID: 11166429

