# Correction: Development of a highly efficient protoplast regeneration and transfection protocol for enhancing CRISPR genome editing of *Brassica carinata*

**DOI:** 10.3389/fpls.2026.1802940

**Published:** 2026-02-17

**Authors:** Frontiers Production Office

**Affiliations:** Frontiers Media SA, Lausanne, Switzerland

**Keywords:** *Brassica carinata*, protoplast regeneration, transfection, oilseed and industrial crop, genome editing

The present addresses “High and Midland Oilseeds Research Program, Ethiopian Institute of Agricultural Research, Addis Ababa, Ethiopia” and “Division of Biotechnology and Plant Health, Norwegian Institute of Bioeconomy Research, Ås, Norway” were incorrectly included as affiliations 2 and 3.

The original version of this article has been updated.

